# The WHO simulation initiative: improving global health partnerships

**DOI:** 10.1186/1747-5341-8-6

**Published:** 2013-07-16

**Authors:** Joseph R Fitchett, Paul G Reidy, Elizabeth J Anderson, Sebastien Forte, Kenrry Chiu

**Affiliations:** 1 Imperial College School of Medicine, London, UK; 2 Royal Hampshire County Hospital, Winchester, UK; 3 Cardiff University School of Medicine, Cardiff, UK; 4 European Central Bank, Frankfurt, Germany; 5 Department of Medicine, McGill University, 845 Sherbrook Street West, Montreal, QC, Canada

## Addressing the problem

Health is unequivocally global. Increasing numbers of students and young professionals in health-allied fields are looking to collaborate and work beyond the confines of their national borders. National governments are committed to improving the health of people across the world, as outlined in an example document “Health is Global: a UK Government strategy 2008-2013” [1], which considers the benefits of health improvement to be reciprocal to all parties involved.

We therefore need appropriately trained human resources to deliver improvements in global public health.

At present, few opportunities exist in the undergraduate medical curriculum to formally develop global public health skills. Medical schools and universities are increasingly establishing modules and indeed degrees at both the undergraduate and postgraduate level focused on global health. These courses are for the most part based on a formula of lectures, tutorials and a research project, and do not necessarily expose students to the skills required for global health diplomacy. The recent WHO Simulation Initiative, outlined in this brief report, looks to redress this gap by providing delegates with an innovative environment to discuss topics of global health importance, gain confidence in public speaking and develop the negotiation skills required to affect change at an institutional level.

Simulation based education is a powerful learning tool. The recent Commission on the Education of Health Professionals for the 21^st^ Century—a global independent initiative [2]—was launched in January 2010 to review medical education across the globe with the aim of “transforming education to strengthen health systems in an interdependent world”. A key recommendation of the commission was for education to adopt transformative learning. This would involve developing the current model built on acquiring knowledge, skills and values, to one where the end goal was furthering leadership attributes and becoming an agent of change. The WHO Simulation initiative is a step in this direction.

## Simulating the WHO World Health Assembly

MonWHO (Montréal World Health Organisation simulation) and EuWHO (European World Health Organisation simulation) were established to provide delegates with firsthand experience of a WHO World Health Assembly. Each delegate was assigned to a UN member state and asked to prepare a position statement prior to the conference. The first EuWHO simulation in 2010 focussed on “the right to health and access to essential medicines and vaccines”. The second EuWHO simulation in 2012 focussed on “population and reproductive health: addressing the unmet need”. The aim was firstly to discuss the member state’s national and regional interests within their respective regional session, and secondly to convene all regions within a plenary session to discuss global interests on the topic. The EuWHO 2012 conference was opened on Friday night (26.10.2012) and addressed by speakers with vastly differing backgrounds, from a WHO representative to the CEO of a non-governmental organisation investing in the education and empowerment of adolescent girls to end poverty. Saturday and Sunday (27 to 28.10.2012) saw the delegations debate, discuss, argue and negotiate to achieve a conference resolution, which represents a youth opinion on what we may need to do to galvanise enthusiasm and bring about tangible change in reproductive health across the world. There were particularly contentious discussions surrounding abortion and traditional cultural practices, as delegates immersed themselves in their roles; arguing points that might be quite contra to their own opinions, and at the same time gaining an appreciation of the difficulties of reconciling such differences in a multilateral context. The Sunday plenary session represented the pinnacle of the weekend as delegates produced a written piece that reflects the consensus views of young people from across the world on the possible solutions to addressing population and reproductive health needs. The EuWHO 2012 Resolution, co-authored by all delegates, is available online (http://www.rsm.ac.uk/euwho/downloads/resolution.pdf).

## What lessons were learned?

Out of 230 delegates at EuWHO 2010, 158 (69%) completed the questionnaires evaluating the simulation. A total of 77% of responders considered the simulation to be relevant or very relevant to their educational needs and 93% of responders believed that the simulation met or exceeded the meeting’s objectives. In addition, 92% of responders rated the content of the introductory lectures as good or very good. Overall, 86% of responders rated EuWHO as good or very good. Considering none of the delegates had any significant experience in formal debate or global health diplomacy, we were delighted to see the majority both settling into their roles & enjoying the experience. Even more encouragingly former delegates have gone on to host similar simulations within their own countries and universities further showing the appeal and utility of such events.

## Overall conclusions

Together with colleagues from the International Federation of Medical Students’. Associations (IFMSA), we are establishing a transnational WHO Simulation. Project to facilitate more simulations. We hope to encourage universities to consider this method of learning, and aim to support one simulation on every continent by the end of 2013.

## Competing interests

The authors declare that they have no competing interests.

## Authors’ contributions

JRF, PGR and EJA worked on the EuHO simulation. SF and KC worked on the MonWHO simulation. JRF wrote the first draft. PGR, EJA, SF and KC reviewed the first draft. All authors read and approved the final manuscript.
